# Profiling Protein Aggregate Size Using Single-Molecule Array Technology

**DOI:** 10.1021/acs.analchem.6c03315

**Published:** 2026-06-25

**Authors:** Dorothea Böken, Yunzhao Wu, Jianli Zhang, Zengjie Xia, Paula Beltran-Lobo, Cara L. Croft, Savinu Weerasekera, Amanda Heslegrave, Henrik Zetterberg, Ashvini Keshavan, Jonathan M. Schott, Maria Jimenez-Sanchez, David C. Duffy, David Klenerman

**Affiliations:** † Yusuf Hamied Department of Chemistry, 2152 University of Cambridge, Cambridge CB2 1EW, U.K.; ‡ UK Dementia Research Institute, University of Cambridge, Cambridge CB2 0AH, U.K.; § 299024 Quanterix Corporation, 900 Middlesex Turnpike, Billerica, Massachusetts 01821, United States; ∥ UCL Queen Square Institute of Neurology, London WC1N 3BG, U.K.; ⊥ UK Dementia Research Institute at University College London, London WC1N 3BG, U.K.; # Hong Kong Center for Neurodegenerative Diseases, Hong Kong 999077, China; ¶ Department of Basic and Clinical Neuroscience, Maurice Wohl Clinical Neuroscience Institute, King’s College London, London SE5 9RX, U.K.; ∇ Centre for Neuroscience, Surgery and Trauma, The Blizard Institute, 4617 Queen Mary University of London, London E1 2AT, U.K.; ○ Department of Psychiatry and Neurochemistry, Institute of Neuroscience and Physiology, The Sahlgrenska Academy at the University of Gothenburg, Mölndal 43180, Sweden; ⧫ Clinical Neurochemistry Laboratory, Sahlgrenska University Hospital, Mölndal 43180, Sweden; †† Wisconsin Alzheimer’s Disease Research Center, University of Wisconsin School of Medicine and Public Health, University of Wisconsin–Madison, Madison, Wisconsin 53792, United States; ‡‡ Department of Pathology and Laboratory Medicine, University of Wisconsin School of Medicine and Public Health, Madison, Wisconsin 53792, United States; §§ Dementia Reseach Centre, Department of Neurodegenerative Disease, UCL Queen Square Institute of Neurology, London WC1N 3BG, U.K.

## Abstract

Protein aggregation is a central feature of many neurodegenerative diseases, yet methods to characterize aggregate size in complex biological samples remain limited. Here, we show that fluorescence intensity from individual single-molecule array (Simoa) microwells encodes size-dependent information beyond conventional digital quantification. Using defined synthetic tau assemblies, we establish that increasing aggregate size produces higher microwell brightness. Applying this technique to human brain homogenate reveals a shift toward larger tau aggregates in Alzheimer’s disease compared to age-matched controls, in agreement with orthogonal measurements by single-molecule super-resolution microscopy. Brightness profiling further captures time-dependent aggregate size increase in a neuronal cell model, demonstrating sensitivity to dynamic changes in aggregation. Although resolution is limited between similarly sized small species, Simoa brightness robustly reports population-level shifts in aggregate size distributions. These findings repurpose a widely used ultrasensitive detection platform to provide high-throughput structural as well as quantitative insight into protein aggregation in biological systems.

## Graphical abstract

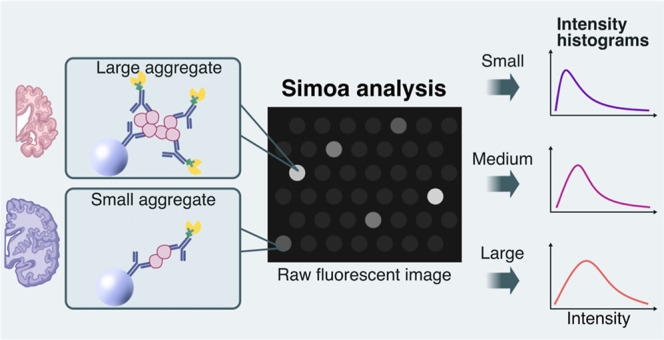

## Introduction

Protein aggregation plays a central role in the pathogenesis and progression of many diseases, particularly neurodegenerative diseases such as Alzheimer’s disease (AD) and Parkinson’s disease (PD).^1,2^ In these conditions, misfolded monomeric proteins form aggregates that lose their physiological functions and may also acquire toxic properties that disrupt cellular homeostasis.^3^ Notably, small (submicron/nanoscopic), soluble oligomers formed at the early stages of aggregation exhibit distinct structural and functional characteristics compared to large, fibrillar aggregates that are often formed at the late stages. Accumulating evidence suggests that these nanoscopic aggregates are the primary cytotoxic species, promoting neuronal death and seeding further aggregation throughout the brain.^4–7^ Importantly, aggregate size distributions vary across pathological stages, highlighting aggregate size as a pathological and potentially diagnostic parameter.^8,9^ Characterizing the size of these aggregates is therefore essential for understanding how protein aggregation drives neurodegenerative disease and for enabling the development of next-generation diagnostics.

Characterization of protein aggregates in biological samples has proven challenging, however, because the aggregates are highly heterogeneous, and conventional bulk assays such as enzyme-linked immunosorbent assay (ELISA) and mass spectrometry lack the resolution to capture size information at the single-aggregate level. While electron microscopy (EM) and atomic force microscopy (AFM) can resolve individual aggregates, their lack of molecular specificity limits their utility in complex biological samples. In addition, protein aggregates often exist at subnanomolar (nM) to picomolar (pM) concentrations in samples such as blood plasma or serum.^10^ Together, these challenges motivate the innovation of single-molecule approaches that not only detect aggregates with high sensitivity, but also retain information about their size and heterogeneity at the single-aggregate level.^11^

Single-molecule pull-down (SiMPull) offers a feasible solution by capturing protein aggregates on antibody-coated coverslips, labeling them with fluorescent secondary antibodies, and imaging them using single-molecule localization microscopy.^12,13^ This super-resolution imaging technique enables direct size and structural characterization of single aggregates below the diffraction limit (∼200 nm) with high molecular specificity and subnanomolar (nM) sensitivity.^13^ However, the long imaging time required for single-molecule localization microscopy limits the throughput of SiMPull.

To overcome this limitation and enable high-throughput single-molecule detection in complex biological samples,^14^ the single-molecule array (Simoa) technique provides a complementary approach, enabling ultrasensitive detection of single proteins or protein aggregates.^10,15^ Simoa captures single protein aggregates in the sample with antibody-conjugated beads. The captured targets are subsequently labeled with a detection antibody and an enzyme to form an immunocomplex on the bead. The beads are then introduced to a microwell array and sealed in individual microwells, within which the enzyme can catalyze the conversion of a fluorogenic substrate. As such, wells containing immunocomplex-bearing beads exhibit fluorescence (“ON”), while empty or nontarget wells remain dark (“OFF”). The proportion of “ON” wells is used to determine aggregate concentration, achieving sensitivities down to the femtomolar (fM) and attomolar (aM) range with high throughput and automation.^15^ Nevertheless, Simoa’s digital output reflects only average aggregate count, but not size, such that samples with equal aggregate numbers but differing sizes yield identical readouts.

Based on the detection principle of Simoa, we hypothesized that larger protein aggregates, containing more epitopes, may bind more detection antibodies and enzymes, thereby generating stronger fluorescent signals in the corresponding microwells. Consequently, the fluorescence intensities of microwells may correlate with the size of individual protein aggregates (Figure 1). In this study, we tested this hypothesis in the context of tau aggregation, a central pathological feature of AD and other tauopathies.^2^ Using our previously developed tau aggregate Simoa assay^10^ based on the pan tau antibody HT7, we used synthetic peptides and peptide-conjugated silica nanoparticles (SiNaPs) to mimic monomeric tau and tau aggregates,^10,16^ respectively, and analyzed their intensity profile. We then applied this approach to test homogenates from post-mortem brain samples from AD patients and healthy controls, comparing fluorescence intensity distributions with aggregate size profiles obtained from super-resolution imaging. With this approach, we aim to establish a quantitative framework linking Simoa fluorescence intensity to protein aggregate size, thereby extending its utility beyond simple aggregate quantification toward size-resolved characterization in complex biological samples.

**Figure 1:**
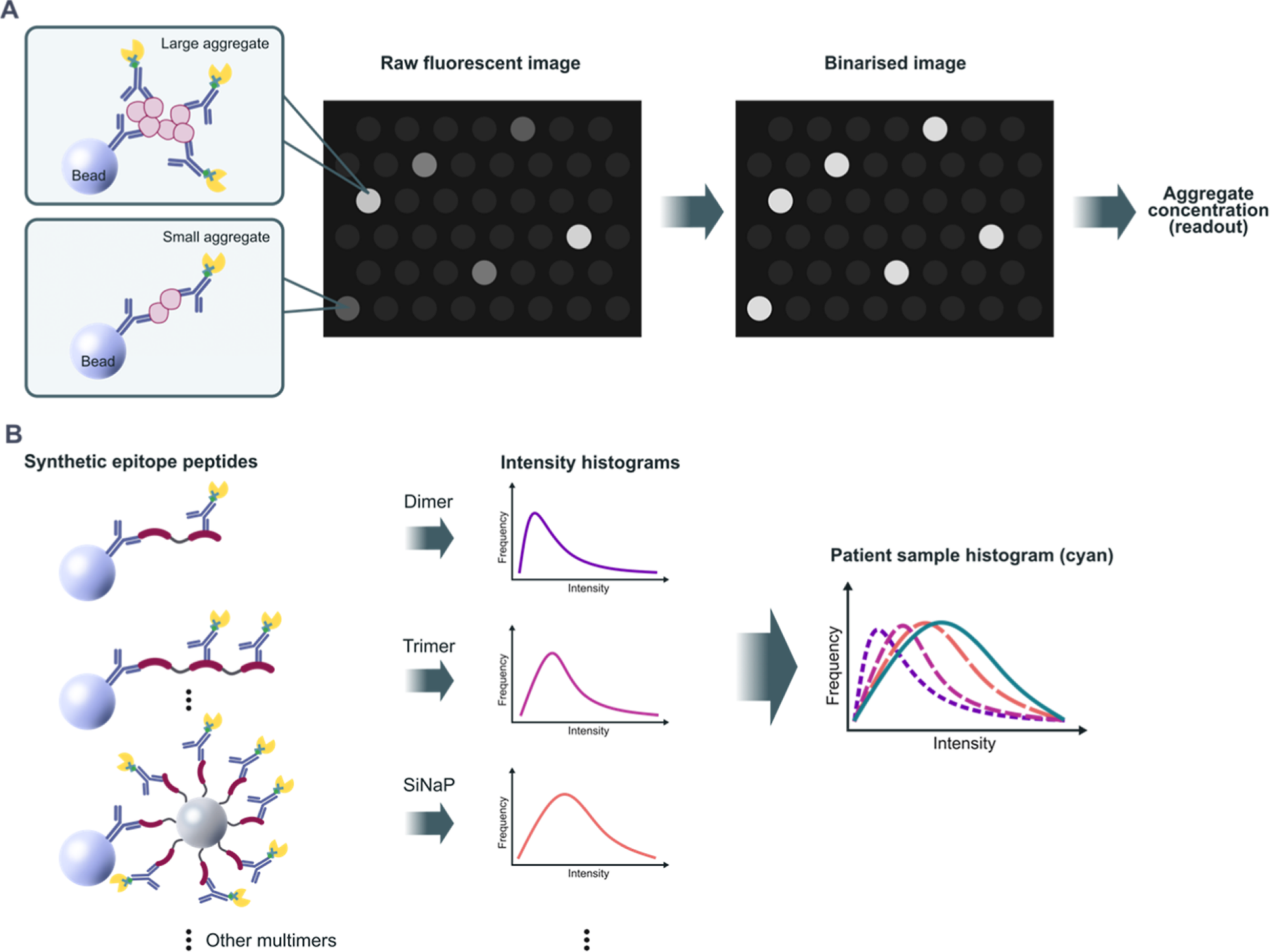
Brightness-based Simoa analysis enables aggregate size profiling. (A) In standard Simoa assays, fluorescent signal from each microwell is binarised, allowing the number of active wells to be used to determine the aggregate concentration. However, the raw fluorescence intensity (brightness) of each individual well is also recorded. Larger aggregates recruit more detection antibodies, resulting in increased enzyme loading and higher microwell fluorescence intensity. (B) Defined synthetic constructs and silica nanoparticle mimetics generate size-dependent intensity distributions, enabling validation and interpretation of aggregate size distributions in biological samples.

## Experimental Section

### Brain Tissue

Post-mortem brain tissue from five donors with AD and five age-matched control donors was obtained from the Edinburgh Brain Bank with the approval from the East of Scotland Research Ethics Service (REC 21/ES/0087). Written informed consent from the donors and/or their relatives was provided as appropriate. Donor characteristics are summarized in Supporting Information Table S2, for which no significant differences were identified between each cohort (AD vs HC).

### Brain Tissue Homogenization

Brain tissue was homogenized following a previously established protocol.^13^ The brain homogenization (BH) buffer was prepared by dissolving one tablet each of phosphatase inhibitor (Merck, Cat. No. 4906845001; 1 tablet/10 mL) and protease inhibitor (Merck, Cat. No. 5892791001; 1 tablet/10 mL) into a solution containing 10 mM Tris-HCl, 0.8 M NaCl, 1 mM EGTA, 0.1% Sarkosyl, and 10% sucrose (pH 7.32). The resulting buffer was filtered through a 0.22 μm syringe filter. Approximately 120 mg of frozen brain tissue and 1.2 mL of ice-cold BH buffer were added to each homogenization tube (Merck, Cat. No. Z763721-50EA). The tube was placed on a VelociRuptor V2Microtube Homogenizer (Scientific Laboratory Supplies, Cat. No. SLS1401) and processed at 4 °C using the following program: (1) 5 m/s for 20 s; (2) 10 s rest; (3) 5 m/s for 20 s. The tube was then centrifuged at 21,000*g* for 20 min, and the supernatant was transferred to a clean 2 mL microcentrifuge tube (Merck, Cat. No. EP0030108450-1EA). Next, 600 μL of ice-cold BH buffer was added to the homogenization tube, and the homogenization process was repeated using the same program. After a second centrifugation at 21,000*g* for 20 min, the resulting supernatant was collected and combined with the first fraction. Total protein concentrations were measured using the BCA assay (ThermoFisher, Cat. No. 23225) according to the manufacturer’s instructions.

### Wolfson CSF Study Plasma Samples

Ethical approval for use of plasma samples was provided by an existing study (Wolfson CSF study 12/0344; NRES London Queen Square, August 2013, PI Schott). Donor characteristics are summarized in Supporting Information Table S3. Participants either provided written informed consent or consultee assent for participation. Participants were recruited at the time of attendance for diagnostic lumbar punctures in the context of workup for cognitive symptoms. Recruitment, plasma sampling, processing and storage until analysis have previously been described.^22^ Plasma samples had undergone 2 freeze–thaw cycles, and were centrifuged for 5 min at 10,000*g* just prior to measurement in this study. Participants were classified as AD biomarker status positive if CSF Aβ42/Aβ40 ratio was ≤0.065 AND p-tau181 >57 pg/mL.

### Preparation of Peptides and Peptide-Conjugated SiNaPs

The HT7-epitope peptides were purchased from Genscript (amino acid sequences are shown in Supporting Information Table S1). Peptide identity and purity were verified by the manufacturer using HPLC and mass spectrometry. The peptides were dissolved in 18.2 MΩ·cm water to a final concentration of 1 mg/mL, aliquoted, and stored at −20 °C. The peptide-conjugated SiNaPs were prepared following previously described protocols.^23^ Briefly, 1 mL of 15 nm 3-aminopropyl­(3-oxobutanoic acid) functionalized SiNaPs (Merck, Cat. No. 660450, estimated concentration 11.47 μM) or 30 nm carboxylated SiNaPs (DiagNano Carboxyl Silica Nanoparticles, CD Bioparticles, Cat. No. DNG-F407, estimated concentration 1.48 μM) were first mixed 1:1 with 18.2 MΩ·cm water. The mixture was centrifuged at 16,900*g* for 1 h at room temperature, and the pellet was resuspended in 2-(*N*-morpholino)­ethanesulfonic acid (MES) buffer (10 mM, pH 5.7). Meanwhile, 1-ethyl-3-(3-(dimethylamino)­propyl) carbodiimide (EDC, ThermoFisher, Cat. No. A35391) and sulfo-*N*-hydroxysuccinimide (sulfo-NHS, ThermoFisher, Cat. No. A39269) were dissolved in cold MES buffer (10 mM, pH 5.7) at 10 and 20 mg/mL, respectively. The EDC and sulfo-NHS solutions were mixed with the SiNaP suspension to achieve final concentrations of 100 nM SiNaP, 400 μM EDC, and 100 μM sulfo-NHS with a total volume of 2 mL. The mixture was sonicated in an ice-water bath for 30 min and centrifuged at 16,900*g* for 1 h at 4 °C. The activated SiNaP pellet was then resuspended in 1 mL of MES buffer and mixed with 50 μL of lysine-(PEG)_8_-modified HT7-binding peptide (Supporting Information Table S1) solution at 1 mM. The reaction mixture was placed on a HulaMixer (ThermoFisher Scientific) and incubated overnight at room temperature. Finally, the reaction mixture was centrifuged at 16,900*g* for 1 h and washed with 1 mL of 1:1 H_2_O/DMSO (v/v). The SiNaP pellet was finally resuspended in 200 μL of 1:1 H_2_O/DMSO (v/v) and stored at −20 °C. The final concentration of the SiNaP suspension was estimated to be 1 μM, assuming no loss during the preparation process.

### Preparation of rAAVs

rAAV2/TM8 ^24^ to express wt human tau (WT-htau0N4R) or pro-aggregant mutant human tau (P301L/S320F-htau0N4R) under the control of the Synapsin promoter was prepared as microscale rAAVs. HEK293T cells (ATCC) were maintained as recommended in DMEM (Gibco, Life technologies) with 10% (v/v) FBS (Gibco, Life Technologies), 2 mM l-glutamine, and 1% (v/v) penicillin/streptomycin (Thermo Fisher Scientific). HEK293T cells were plated in 15 cm dishes in 25 mL media 24 h before transfection of transgene plasmid with TM8 capsid (AAV2/8-3Y) and pHelper using polyethylenimine linear (Polysciences) at 60–80% confluency. Twenty-four h following transfection, the media was changed to serum-free Optimem (Gibco, Life Technologies) with 1% v/v penicillin/streptomycin. The microscale virus was harvested 48 h after switching to Optimem by centrifuging the media at 500 g for 5 min and collecting the supernatant. The supernatant was concentrated by centrifugation at 4000 rpm using Amicon 15 100,000 MWCO concentration units (Millipore, UFC910024). Genomic titers (viral genomes (VGs) per mL) were confirmed via quantitative real time PCR (qPCR) with probes against the ITRs as previously described by Goodwin et al.^24^ Concentrated media containing secreted rAAVs (microscale virus) were then applied to primary neurons by adding rAAVs directly into the culture medium at 2.5 × 10^10^ VG/mL.

### Primary Mouse Cortical Neurons

Primary mouse cortical neurons were prepared from the cortex of wild type CD1 embryos at E16.5 as described in Yang et al.^25^ Briefly, brains were harvested in ice-cold dissection buffer containing 1 mM HEPES in HBSS, the meninges removed, and the cerebral cortices isolated. Following mechanical dissociation, cells were seeded at a density of 250.000 cells/well in 12-well plates precoated with 10 μg/mL poly-d-lysine (Sigma-Aldrich, P7280) and cultured in Neurobasal medium (Gibco, 21103049) supplemented with 2% B27 (Gibco, 17504044), GlutaMAX (Gibco, 35050061) and penicillin (100 U/mL) and streptomycin (100 μg/mL; Gibco, 15140122). Cells were placed in a humidified incubator at 37 °C in 5% CO_2_. At 6 DIV, neurons were transduced with adeno-associated viruses (AAVs) to express wt (AAV2/TM8-WT-htau0N4R) or mutant (AAV2/TM8-P301L/S320F-htau0N4R) human tau at 2.5 × 10^10^ viral genome (VG)/mL. Nontransduced cells served as a negative control. Cell culture media was collected at 8 and 14 DIV and centrifuged at 1000*g* for 5 min at 4 °C. Each experiment is defined by an independent neuronal preparation and included two technical replicates (two wells) per time point and viral construct. All conditions were grown in three biological replicates, each including two technical replicates.

### Simoa Bead Conjugation

The Simoa bead conjugation was performed according to the manufacturer’s instructions (Quanterix Corporation). The tau monoclonal antibody HT7 (ThermoFisher, Cat. No. MN1000) was buffer-exchanged into 25 mM 2-(*N*-morpholino)­ethanesulfonic acid (MES) buffer (pH 5.0) using an AmiconUltra-0.5 centrifugal filter (Merck, Cat. No. UFC500396, molecular weight cutoff 50 kDa) and diluted to 0.2 mg/mL. The 488-dyed singleplex assay beads (Quanterix, Cat. No. 104006) were washed three times with 0.01 M NaOH and twice with 18.2 MΩ·cm water with the aid of a magnetic rack (Cytiva, Cat. No. MagRack 6), and resuspended in 280 μL of 25 mM MES buffer to a concentration of 1.5 × 10^9^ beads/mL and kept on ice until use. Meanwhile, 1-ethyl-3-(3-(dimethylamino)­propyl)­carbodiimide (EDC, ThermoFisher, Cat. No. A35391) and *N*-hydroxysulfosuccinimide (sulfo-NHS, ThermoFisher, Cat. No. A39269) were dissolved in ice-cold 25 mM MES buffer at 10 and 40 mg/mL, respectively. Subsequently, 4.5 μL of EDC and 15 μL of sulfo-NHS solutions were immediately introduced to the bead suspension. The bead suspension was placed on a rotational mixer for 30 min at 4 °C. The activated beads were washed once with cold 25 mM MES buffer, resuspended with 300 μL of buffer-exchanged antibody, and placed on the rotational mixer for another 30 min at room temperature. The antibody-conjugated beads were then washed twice with Bead Wash Buffer (Quanterix, Cat. No. 101354) and blocked with Bead Blocking Buffer (Quanterix, Cat. No. 101354) for 40 min on the rotational mixer at room temperature. The blocked beads were washed once with Bead Wash Buffer and once with Bead Diluent (Quanterix, Cat. No. 101362). Finally, the washed beads were resuspended in 300 μL of Bead Diluent and kept at 4 °C until use.

### Simoa Assay

Simoa assays were performed using the standard three-step protocol as previously described.^10^ Briefly, synthetic peptides, SiNaPs, or patient brain homogenates were diluted to the indicated concentrations using Tau 2.0 Sample Diluent (Quanterix, Cat. No. 103847) and loaded into a 96-well plate (100 μL/well). The Simoa bead suspension was diluted to a working concentration of 2 × 10^7^ beads/mL and added to each well (25 μL/well). The plate was placed on a shaking incubator (Quanterix) and incubated at 30 °C for 30 min at 800 rpm. Subsequently, the plate was washed using a microplate washer (Quanterix), followed by the addition of 100 μL of biotinylated HT7 antibody (ThermoFisher, Cat. No. MN1000B) at 0.3 μg/mL in Homebrew Sample/Detector Diluent (Quanterix, Cat. No. 101359) to each well. The plate was incubated for 10 min on a shaking incubator at 800 rpm and then washed again. Next, 100 μL of streptavidin-β-galactosidase fusion protein (SBG) at 150 pM in SBG Diluent (Quanterix, Cat. No. 100376) was added to each well. After a final 10 min incubation at 800 rpm, the plate was washed, dried for 10 min, and loaded onto the Simoa SR-X analyzer for analysis.

For analysis of the ADAPT cohort, assays were performed on the Simoa HD-X analyzer (Quanterix, Cat. No. 103385) using an automated three-step protocol. Capture beads (Simoa 488-dyed Singleplex Beads, Quanterix, Cat. No. 104006) were conjugated to antibodies against tau (HT7, Invitrogen, Cat. No. MN1000), phospho-tau (AT8, Invitrogen, Cat. No. MN1020), α-synuclein (4B12, BioLegend, Cat. No. 807801), or Aβ (6E10, BioLegend, Cat. No. 803007), with corresponding biotinylated detector antibodies prepared in General Detector & Sample Diluent (Quanterix, Cat. No. 101359). Beads were diluted to ∼2 × 10^7^ beads/mL in Bead Diluent Buffer (Quanterix, Cat. No. 101362), and SBG was prepared at 150 pM in SBG Diluent. Samples were diluted in Lysate Diluent A (Quanterix, Cat. No. 103358) and loaded into 96-well plates. The HD-X instrument automatically performed bead-sample incubation (30 min), detector incubation (5 min), SBG incubation (5 min), washing, resuspension in RGP reagent, array loading, imaging, and calculation of average enzyme per bead (AEB) values.

### SiMPull Coverslip Preparation

The SiMPull coverslips were prepared as previously reported.^13^ Glass coverslips (VWR, Cat. No. MENZBC026076AC40) were first cleaned in an ultrasonic bath with 18.2 MΩ·cm water for 10 min, followed by two additional sonication steps in acetone and methanol, respectively. The coverslips were then sonicated in 1 M KOH for 20 min, rinsed sequentially with methanol and water, dried with nitrogen, and subjected to argon plasma cleaning for 15 min using a plasma cleaner (PDC-002, Harrick Plasma). Following plasma treatment, the coverslips were silanized using a 5:3:100 mixture of acetic acid (Merck, Cat. No. 45726), 3-aminopropyl triethoxysilane (Fisher Scientific UK, Cat. No. 10677502), and methanol. The silanization was performed with two 60 s sonication cycles, separated by a 10 min rest. The coverslips were then rinsed with water and methanol, dried with nitrogen, and fitted with a 50-well polydimethylsiloxane (PDMS) gasket (Merck, Cat. No. GBL103250) centered on the surface. To each well, 9 μL of an aqueous 100:1 mixture of methoxy-PEG-succinimidyl valerate (110 mg/mL, Laysan Bio Inc., Cat. No. MPEG-SVA-5000) and biotin-PEG-succinimidyl valerate (100 mg/mL, Laysan Bio Inc., Cat. No. Biotin-PEG-SVA-5000) and 1 μL of 1 M NaHCO_3_ (pH 8.5) were added for surface passivation. The coverslips were incubated overnight at room temperature in a humidity chamber, rinsed with 18.2 MΩ·cm water, and dried with nitrogen. A second passivation step was then performed by introducing 9 μL of 10 mg/mL methyl-PEG4-NHS ester (Thermo Fisher, Cat. No. 22341) and 1 μL of 1 M NaHCO_3_ to each well. The coverslips were again incubated overnight, washed with 18.2 MΩ·cm water, dried with nitrogen, and stored in a vacuum desiccator at −20 °C until use.

### SiMPull Assay

The DNA-PAINT SiMPull assay was performed as previously described.^26^ The SiMPull coverslips were first removed from the freezer and allowed to equilibrate to room temperature. To each well, 10 μL of 0.2 mg/mL neutravidin solution (ThermoFisher, Cat. No. 31000) in PBST (phosphate buffered saline (PBS, ThermoFisher, Gibco, Cat. No. 10010023) supplemented with 0.5% Tween-20 (Merck, Cat. No. P1379-100 ML)) was introduced. The coverslip was incubated in a humidity chamber for 10 min at room temperature, followed by two washes with PBST and one wash with PBS containing 1% Tween-20. Next, 10 μL of 10 nM biotinylated HT7 capture antibody (ThermoFisher, Cat. No. MN1000B) in a blocking buffer (PBS supplemented with 1 mg/mL bovine serum albumin (BSA, Thermo Fisher, Cat. No. B14)) was introduced to each well. The coverslip was again incubated for 10 min at room temperature and washed three times (twice with PBST and once with PBS + 1% Tween-20). The coverslip was then blocked by the blocking buffer for 10 min and washed once with PBST. The samples were then added to the wells, and the coverslip was incubated for 1 h at room temperature. After incubation, each well was washed three times (twice with PBST and once with PBS + 1% Tween-20), and 10 μL of 1 nM fluorescently labeled detection antibody (HT7-AlexaFluor647, ThermoFisher, Cat. No. MN1000) in blocking buffer was added. The coverslip was incubated for 15 min and washed three times (twice with PBST and once with PBS + 1% Tween-20). Finally, all wells were filled with 10 μL of PBS before imaging.

### Super-Resolution Imaging and Analysis

The super-resolution imaging was performed with a home-built total internal reflection fluorescence (TIRF) microscope. The SiMPull coverslip was placed on an inverted Ti-2 Eclipse microscope body (Nikon) fitted with a 1.49 N.A., 60× TIRF objective (Apo TIRF, Nikon) and was illuminated by a 638 nm laser (Cobolt 06-MLD-638, HÜBNER) operating at 190 mW output in the TIRF mode. The fluorescence was collected by the same objective, passed through a quad-band dichroic beam splitter (Di01-R405/488/561/635-25x36, Semrock), and was cleaned up by an emission filter (BLP01-635R-25x36, Semrock) before being recorded by an EMCCD camera (Evolve 512, Photometrics) operating in frame-transfer mode (EM gain = 6.3 electrons/ADU and 250 ADU/photon). The pixel size of the camera was measured to be 107 nm, each image was 512 × 512 pixels. The microscope and peripheral devices were controlled by the MicroManager 1.4 software and images were acquired automatically using a custom BeanShell script. Lasers were set to maximum power (190 mW, 638 nm), with three fields of view imaged per well, each for typically 7000 frames of 100 ms exposure.

Super-resolution images were reconstructed using the Picasso package.^27^ Briefly, after discarding the first 100 frames, localizations were identified, fitted, and then corrected for microscope drift using redundant cross-correlation. Localizations were then filtered for precision <30 nm. To obtain aggregate clusters, DBSCAN was applied using the scitkit-learn package^28^ (radius of 0.3 (∼35 nm) and minimum density of 5). Each aggregate was then measured using a combination of scikit-image^29^ for basic region properties such as perimeter, area, and eccentricity, and SKAN^30^ for skeletonized length whereby the length of each aggregate is reported as the summed branch distance. Finally, super-resolved images were rendered using the inbuilt Picasso^27^ functionality.

### Data Analysis

The Simoa intensity profile of each well was extracted from the raw images obtained by the Simoa SR-X analyzer using custom software. The brightness data were analyzed using a home-written Python script. Briefly, the modified AEB (mAEB) values were calculated at different thresholds using the following equation: mAEB = −ln­(1 – *f*_ON_), where *f*_ON_ is the fraction of microwells with fluorescent intensity above a given threshold. The default intensity thresholds for the SR-X and HD-X analyzers were 110 and 40, respectively. For serum samples, the optimal intensity threshold for each biomarker was defined as the value maximizing the AUC of the ROC curve, thereby providing the best discrimination between AD patients and controls. Accordingly, the optimal thresholds for the HT7, AT8, 6E10, and 4B12 assays were determined to be 4400, 3010, 290, and 50, respectively. To evaluate the combination of HT7 and AT8, two logistic regression models were developed using mAEB values obtained at either the default or optimal cut-offs. Performance was assessed using a bootstrap method: the data set was randomly split into training (70%) and testing (30%) sets over 100 iterations, from which mean ROC curves were generated.

## Results

### Brightness-Based Analysis Distinguishes Tau Assemblies of Increasing Size Using Defined-Size Reference Constructs

We first tested whether Simoa microwell brightness reflects aggregate size using synthetic tau assemblies of defined size. For this assessment, we focused on a well-characterized tau antibody, HT7. A previously validated HT7 Simoa aggregate assay was used throughout, employing the same antibody for capture and detection to ensure selective detection of multimeric tau species.^10^ Under these conditions, monomeric tau is not expected to generate signal, as only a single HT7 binding site is present.

A series of synthetic reference constructs of defined size was designed to test the principle that increasing aggregate size results in increased microwell brightness. These constructs included synthetic peptides comprising one to five HT7 epitopes joined by a PEG linker, mimicking monomeric to pentameric tau assemblies, as well as larger aggregates mimicked by conjugating the HT7 epitope to silica nanoparticles of defined size (15 and 30 nm diameter). Together, these constructs span a range of aggregate sizes while maintaining controlled molecular composition and a defined number of HT7 epitopes.

Using these defined-size constructs, we observed a progressive increase in detection sensitivity with increasing aggregate size (Figure 2A and Supporting Information Table S1). While the monomeric construct remained at background levels across all concentrations tested, assemblies containing multiple HT7 epitopes produced detectable signal at progressively lower concentrations. This behavior indicates that larger aggregates can be detected at lower abundance. Consistent with this observation, applying higher brightness thresholds (i.e., restricting analysis to higher-intensity microwells) modestly improved the limit of detection in silica nanoparticle-based constructs, as this adjustment reduces background signal (Supporting Information Figure S1A). However, while more stringent thresholds enhance detection at low signal levels, they also reduce the number of detectable events.

**Figure 2:**
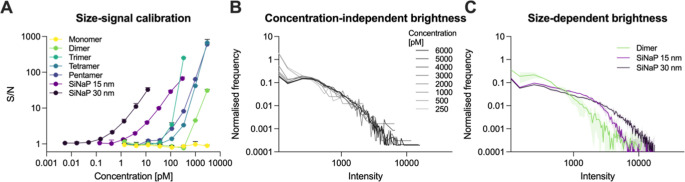
Brightness-based validation of tau aggregate size using defined-size reference constructs. (A) Calibration curves showing signal-to-background ratios for synthetic aggregate-mimicking constructs of increasing size, including peptides containing one to five HT7 epitopes and HT7 epitope-conjugated silica nanoparticles (SiNaPs; 15 and 30 nm diameter). Data represent mean ± SD from *n* = 2 technical replicates. (B) Intensity histograms for the dimeric peptide measured across a range of concentrations. (C) Comparison of brightness distributions for selected constructs at matched AEB levels, including the dimeric peptide and 15 and 30 nm SiNaPs. Data represent mean ± SD from *n* = 3 technical replicates.

To determine whether brightness distributions depend on analyte concentration, intensity histograms were generated for the dimeric construct across a range of concentrations (Figure 2B). Within a low average enzyme per bead (AEB) regime, where >95% of beads are expected to capture either zero or one analyte (based on Poisson statistics), the position and shape of the intensity distributions remained stable across concentrations. This indicates that, within the low AEB regime, microwell brightness is largely independent of analyte concentration and reflects properties of the captured assemblies rather than analyte abundance.

Analysis of the brightness distributions revealed construct-dependent differences in signal intensity (Figure 2C). The monomeric peptide exhibited a narrow distribution centered near background levels, whereas the dimer showed a broad distribution despite only a single detection antibody being able to bind per captured analyte. This variability likely reflects heterogeneity in enzyme labeling and enzymatic turnover^17^ rather than differences in aggregate size. Assemblies containing three to five HT7 epitopes produced overlapping intensity distributions (Supporting Information Figure S1B), indicating that Simoa brightness cannot reliably resolve closely spaced oligomeric sizes in this regime. In contrast, silica nanoparticle-based constructs produced substantially higher intensities, with clear separation between the 15 and 30 nm assemblies at higher intensities (Figure 2C, PERMANOVA *F* = 8.902, *R* ^2^ = 0.748, *p* = 0.0107).

Together, these results demonstrate that Simoa microwell brightness provides size-dependent information for tau aggregates and can robustly distinguish broadly defined classes of aggregate size. While brightness measurements are not suited for fine-grained resolution of closely related small oligomeric species, they provide a powerful proof-of-principle approach for extracting aggregate size information at the single-molecule level.

### Brightness Profiling Reveals a Shift toward Higher-Intensity Tau Aggregates in Alzheimer’s Disease Brain Homogenate Compared with Healthy Controls

Having established that Simoa microwell brightness provides size-dependent information for defined tau assemblies, we next applied this approach to a more biologically relevant sample type, human brain homogenate. Post-mortem brain tissue homogenates were prepared in a low-detergent extraction buffer (10 mM Tris-HCl, 0.8 M NaCl, 1 mM EGTA, 0.1% Sarkosyl, and 10% sucrose), enriching for soluble tau species. Post-mortem brain tissue homogenates were obtained from individuals with AD (Braak stage VI, BA 6/8, *n* = 5) and from age- and sex-matched neurologically healthy controls (Braak stage 0, BA 6/8, *n* = 5) (Figure 3A, donor information in Supporting Information Table S2).

**Figure 3:**
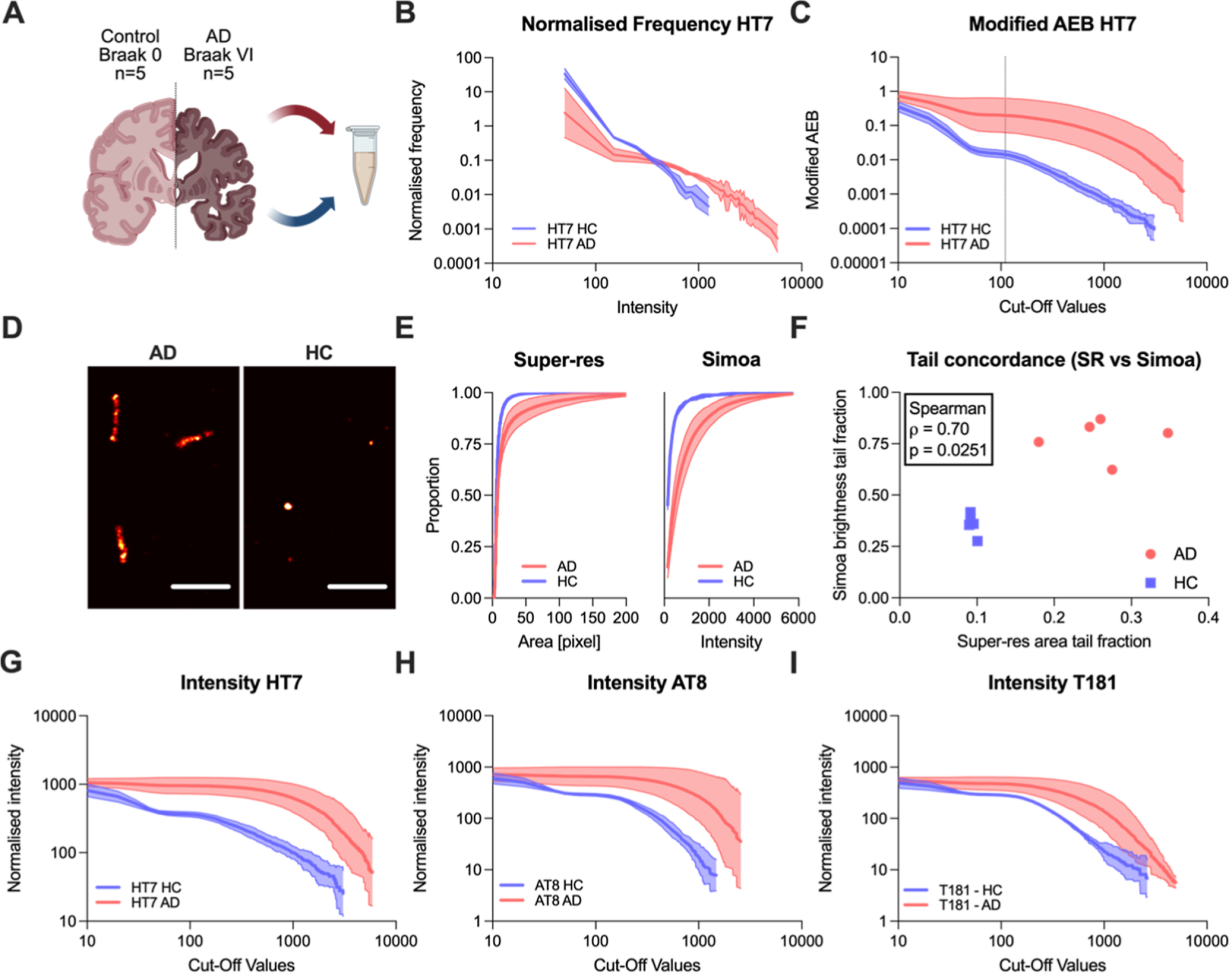
Brightness profiling of tau aggregates in human brain homogenate and comparison with super-resolution microscopy. All curves represent mean ± SD across patients (*n* = 5 AD, *n* = 5 HC). (A) Schematic of post-mortem brain tissue samples used in the study. (B) Brightness distributions of tau aggregates measured by the HT7 Simoa aggregate assay in AD and HC brain homogenates, normalized to the total number of positive beads per sample. (C) Modified AEB analysis of HT7 Simoa measurements using progressively increased intensity thresholds to redefine positive microwells (standard threshold SR-X = 110). Raising the cutoff selectively enriches for brighter microwells. The two curves were compared using a nonparametric two-sample permutation test for functional data. The test statistic was the integrated squared difference between the two group mean curves. Statistical significance was assessed by exact permutation of group labels across replicate curves (*p* = 0.00794). (D) Representative super-resolution microscopy images of tau aggregates captured from control and AD brain homogenates. Scale bar = 1000 nm. (E) Cumulative distributions of aggregate area measured by super-resolution microscopy and of intensity measured by Simoa for AD and control samples. (F) Comparison of tail fractions derived from super-resolution (SR) microscopy and Simoa brightness measurements. The super-resolution tail fraction represents the fraction of aggregates exceeding a size threshold defined by the upper decile of the control distribution, while the Simoa tail fraction represents the fraction of total signal contributed by high-intensity microwells exceeding the corresponding control-defined brightness threshold. Each point represents one brain homogenate sample. (G–I) Thresholded cumulative brightness profiles for tau aggregates detected using phosphorylation-specific Simoa assays targeting AT8 (S202/T205) and T181 epitopes. Data represent mean ± SD from *n* = 5 patients per group.

Tau aggregates were measured using the HT7 Simoa aggregate assay, and brightness histograms were generated for each sample. To enable comparison of brightness distributions independent of aggregate burden, histograms were normalized to the total number of positive beads per sample. This analysis revealed a clear redistribution of signal intensity in AD brain samples compared to controls, showing an enrichment of higher-intensity (“brighter”) microwells in the upper tail of the distribution (Figure 3B).

Given that the conventional Simoa analysis classifies all microwells with intensities above a fixed threshold (SR-X: intensity of 110) as positive, this shift in the upper tail of the distribution is not captured by the standard AEB metrics. We therefore explored whether redefining the intensity threshold to selectively capture brighter microwells could enhance separation between samples. By progressively increasing the intensity cutoff, thereby selectively enriching for higher brightness/larger aggregates, we observed improved separation between AD and control samples, consistent with an increased contribution of larger tau aggregates in AD brain homogenate (Figure 3C).

### Brightness-Based Simoa Metrics Are Consistent with Aggregate Size Distributions Measured by Super-Resolution Microscopy

To assess whether these brightness-based differences reflect shifts in aggregate size, we analyzed the same brain homogenate samples using single-molecule super-resolution microscopy (Figure 3D–F). Super-resolution imaging enabled direct measurement of individual tau aggregate dimensions in the same samples, allowing aggregate size distributions to be compared between AD and control groups between the two methods.

Analysis of aggregate area distributions revealed a rightward shift toward larger tau aggregates in AD samples compared to controls (Figure 3E), indicating an increased contribution of large aggregates to the overall population. The overall shape of the cumulative area distributions closely resembled the brightness distributions obtained by Simoa analysis of the same samples.

To enable a quantitative comparison between both methods, tail fractions were defined for super-resolution and Simoa measurements using thresholds corresponding to the upper decile of the respective control distributions, based on aggregate size and microwell brightness, respectively. Across matched samples, enrichment of large aggregates measured by super-resolution microscopy was accompanied by an increased contribution of bright wells in Simoa measurements (Figure 3F). These two independently derived tail fractions showed strong concordance across samples (Spearman ρ = 0.70, *p* = 0.025).

Together, these analyses indicate that brightness-based Simoa metrics capture population-level shifts in tau aggregate size that are consistent with direct measurements obtained by super-resolution microscopy.

### Phosphorylation-Dependent Differences in Tau Aggregate Brightness Profiles

Tau hyperphosphorylation is a central pathological hallmark of AD and other tauopathies, and is closely linked to pathological tau aggregation.^18^ Having established brightness-based differences in tau aggregate populations using the pan-tau antibody HT7, we next examined whether these shifts differed across tau aggregates defined by phosphorylation state.

To this end, tau aggregates in the same brain homogenates were analyzed using Simoa assays detecting tau aggregates phosphorylated at S202/T205 (AT8) and T181, and brightness distributions were generated for each antibody (Figure 3G–I).

To quantify differences in the upper tail of the brightness distributions, we calculated the normalized cumulative brightness above a range of increasing intensity thresholds. For each sample, brightness histograms were first normalized to the total number of positive beads, and the cumulative signal contributed by events exceeding a given intensity cutoff was then determined. This approach enables comparison of high-brightness signal contributions across samples and antibodies without relying on a single fixed threshold.

Using this thresholded cumulative brightness analysis, AD brain samples exhibited a greater contribution of brighter, higher-intensity tau and p-tau aggregates compared to controls. Moreover, differences in the shape and threshold-dependence of the cumulative brightness profiles were observed between total tau (HT7) and phosphorylation-specific tau aggregate populations (AT8 and T181), indicating phosphorylation-dependent differences in aggregate brightness distributions.

### Brightness Profiling of Protein Aggregates across Biological Sample Types

Having established that Simoa microwell brightness reflects aggregate size in defined reference constructs and brain homogenate, we next explored whether brightness profiling remains informative across additional biological sample types (Figure 4A). Blood represents a clinically relevant but challenging matrix due to the low abundance of aggregate species and the presence of abundant background proteins. In plasma samples from symptomatic individuals with CSF-confirmed clinically diagnosed AD (*n* = 129) and non-AD (*n* = 124) from the Wolfson CSF cohort (donor information in Supporting Information Table S3), aggregate measurements showed only modest differences between groups across assays (Supporting Information Figure S2A–D). Although application of brightness-based thresholds resulted in a modest improvement of the classification performance (HT7 + AT8, mean AUC_default_ = 0.49, AUC_cutoff_ = 0.57), the discriminatory performance was limited, indicating that, in this context, disease-associated differences in aggregate size are subtle and not readily captured by brightness-based analysis (Figure 4B). Interestingly, despite the limited diagnostic discrimination, clear differences in absolute brightness distributions were observed between aggregate assays. HT7-positive tau aggregates consistently exhibited substantially higher brightness values than AT8-, amyloid-β-, or α-synuclein-positive aggregates in both AD and control samples (Figure 4C), suggesting that tau aggregates detected by the HT7 assay may comprise larger or more epitope-dense assemblies than the other aggregate species analyzed.

**Figure 4:**
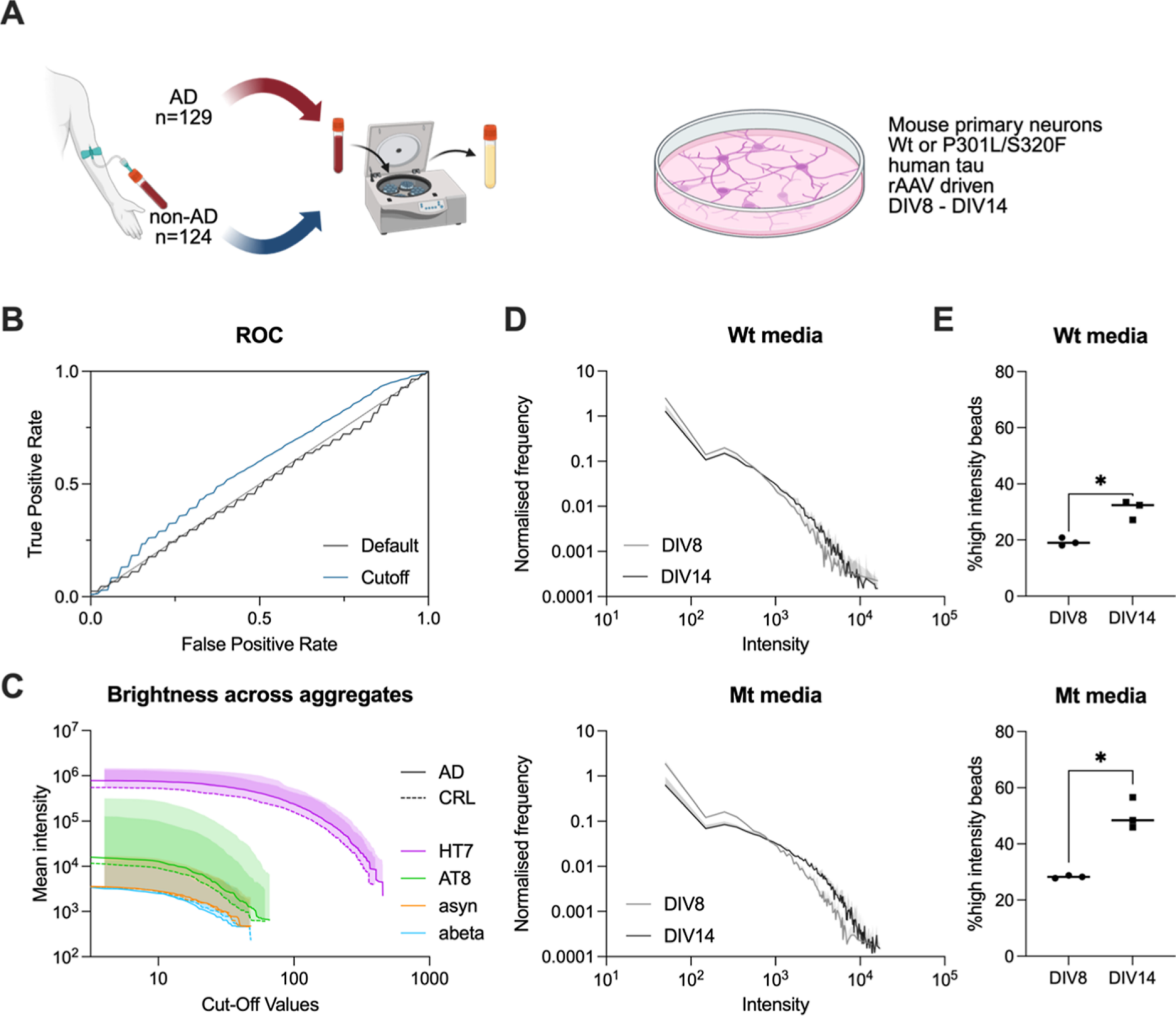
Brightness profiling of protein aggregates across biological sample types. (A) Schematic illustrating sample types analyzed, including human blood samples and conditioned media from primary neuron cultures. (B) Receiver operating characteristic (ROC) curves for aggregate measurements (AT8 + HT7 tau aggregates) in blood samples, using the default and optimized intensity cutoff. (C) Brightness distributions of protein aggregates measured in blood samples using Simoa assays targeting total tau (HT7), phosphorylated tau (AT8), amyloid-β (Aβ), and α-synuclein (α-syn). Curves represent mean ± SD across individuals. (D) Brightness distributions of tau aggregates in conditioned media from primary mouse neurons overexpressing aggregation-prone or wildtype (wt) human tau at different time points (DIV8 and DIV14). Data represent mean ± SD from *n* = 3 biological replicates. (E) Percentage of bright aggregates (well intensity >1000). Welch’s *t*-test, *: *p* < 0.05.

To further assess the behavior of brightness profiling in a controlled biological system, we analyzed conditioned media from primary mouse neurons expressing rAAV-driven wild-type (WT) human tau or an aggregation-prone mutant (P301L/S320F). Brightness distributions revealed a small time-dependent increase in high-intensity signal between 8 and 14 days in vitro (DIV8-DIV14), consistent with the emergence of larger aggregate assemblies over time (Figure 4D). To quantify this shift, we calculated the proportion of aggregates exceeding an intensity threshold of 1000 A.U. (Figure 4E), representing the fraction of brighter (larger) tau aggregates. This proportion increased significantly over time in both WT tau (19.29% at DIV8 vs 31.04% at DIV14, Welch’s *t*-test *p* = 0.0157) and mutant tau (28.26% at DIV8 vs 50.32% at DIV14, *p* = 0.0197). The increase was more pronounced in neurons expressing mutant tau, indicating enhanced aggregation and a greater enrichment of larger species compared to WT tau. Importantly, untransduced control neurons did not show a comparable increase over time and exhibited markedly lower overall signal intensity (Supporting Information Figure S3A, B). These findings demonstrate that brightness-based Simoa analysis is capable of capturing dynamic changes in aggregate size in biologically relevant systems, particularly in contexts where aggregate growth is pronounced. Notably, the ability to detect and characterize aggregates directly in conditioned media enables noninvasive analysis of extracellular tau aggregates, allowing dynamic monitoring of aggregate release without disrupting the cellular system.

## Discussion

In this study, we have demonstrated that fluorescence intensity measurements obtained from individual Simoa microwells contain information beyond conventional digital aggregate quantification and can be leveraged to profile protein aggregate size distributions. Using defined synthetic reference constructs, we show that increasing aggregate size is associated with increased microwell brightness, establishing a direct relationship between Simoa signal intensity and aggregate size. Extending this approach to human brain tissue revealed that brightness-based Simoa metrics capture disease-associated shifts in tau aggregate populations that are consistent with orthogonal measurements obtained by single-molecule super-resolution microscopy. Collectively, these findings expand the utility of Simoa from a digital counting platform to a higher-throughput method for size-resolved aggregate characterization in complex biological samples.

The use of defined synthetic tau reference constructs spanning a range of sizes, including multivalent HT7 epitope peptides and silica nanoparticle-based aggregate mimetics, provided controlled validation of the relationship between Simoa brightness and aggregate size. Increasing construct size was associated with progressively enhanced detection sensitivity and elevated microwell brightness, supporting the principle that larger assemblies generate stronger Simoa signals through increased antibody and enzyme loading. However, similarly sized oligomeric constructs exhibited overlapping brightness distributions, indicating that Simoa brightness lacks sufficient resolution to distinguish fine differences between small aggregates. Importantly, this relationship was further supported in biological samples by the strong concordance between brightness-derived tail fractions and orthogonal aggregate size measurements obtained by single-molecule super-resolution microscopy in matched human brain homogenates. These findings support the use of brightness-based Simoa analysis as a population-level proxy for detecting broader shifts in aggregate size distributions.

Application of brightness-based Simoa profiling to post-mortem human brain homogenates revealed a clear enrichment of higher-intensity tau aggregates in AD samples relative to controls, consistent with a shift toward larger aggregates in disease. These findings align with previous observations that tau aggregation leads to the formation of increasingly larger and structurally complex assemblies during disease progression. The ability of brightness-based Simoa analysis to detect this shift demonstrates that the approach is sufficiently sensitive to capture biologically meaningful shifts in aggregate size distributions in heterogeneous biological samples. Moreover, the differing brightness profiles observed across phosphorylation-specific tau aggregate assays suggest that distinct post-translationally modified tau species may occupy different size distributions, further highlighting the molecular heterogeneity of pathological tau aggregates in AD brain tissue. Consistent with this, brightness profiling in a neuronal cell model of tauopathy revealed a pronounced increase in high-intensity tau aggregates at later stages of aggregation, demonstrating that the approach can capture dynamic changes in aggregate size over time in living systems. In addition, this approach may be extendable to other disease-relevant biofluids, such as cerebrospinal fluid (CSF), although matrix-specific optimization will be required.

Despite these promising findings, several limitations should be considered. Most notably, the overlapping brightness distributions observed for similarly sized synthetic constructs indicate that the approach lacks sufficient resolution to distinguish subtle differences between similarly sized aggregate species. This limited resolution and overall low aggregate concentration in plasma may explain why brightness thresholding did not substantially improve diagnostic discrimination in blood samples, where disease-associated differences are likely to be smaller and more subtle than those observed in post-mortem brain tissue. Furthermore, the non-AD comparison group in the plasma cohort was heterogeneous and included individuals with other neurodegenerative diseases rather than exclusively neurologically healthy controls, which may have further reduced discrimination between groups. In addition, microwell brightness is influenced by factors beyond aggregate dimensions, including epitope density, antibody accessibility, assay-specific labeling efficiency, and stochastic variation in enzyme activity.^17^ Consequently, intensity distributions likely represent a convolution of multiple aggregate and assay properties, making it challenging to deconvolute discrete aggregate species solely from brightness profiles. Accordingly, brightness-based Simoa analysis is best interpreted as a comparative population-level metric that is most sensitive to pronounced shifts in aggregate size distributions. Importantly, this bias toward detecting pronounced size differences may be advantageous in other biological contexts. Future studies may further standardize this approach to reduce assay-derived variability and improve aggregate size resolution. Strategies such as site-specific antibody labeling, defined antibody-enzyme stoichiometry, and precise control of incubation and bead loading conditions may narrow non-size-related brightness variation. In addition, calibration standards with defined sizes and epitope densities, together with computational deconvolution, may help establish more quantitative relationships between microwell brightness and aggregate size.

The relationship between signal readout and aggregate size is also important when comparing the present method with other ultrasensitive immunoassay platforms, such as Olink^19^ and nucleic acid-linked immuno-sandwich assay (NULISA).^20^ These platforms convert antibody-based protein recognition into qPCR- or next-generation sequencing (NGS)-quantified nucleic acid readouts, and aggregate signals may not scale proportionally with aggregate size and are generally measured as bulk signals without single-aggregate resolution. Therefore, the present concept may not be directly transferable to standard Olink or NULISA workflows, but could be adaptable to platforms in which signal amplification remains linked to the number of detection probes bound to an individual captured target, such as rolling circle amplification (RCA)-based assays.

Beyond tau aggregation, brightness-based Simoa analysis may be applicable to a wider range of biological assemblies. Many cellular processes involve the formation of higher-order protein complexes, including large signaling assemblies such as inflammasomes (e.g., ASC specks), phase-separated condensates, and viral particles. In such contexts, the ability to selectively interrogate high-intensity microwells using elevated brightness thresholds could enable preferential detection of rare, large assemblies within complex mixtures. This capability raises the possibility of extending Simoa toward the direct detection of large protein complexes or even nanoscale particles such as viruses without the need for amplification, provided sufficient epitope density and assay sensitivity. More generally, brightness-based analysis may offer a means to distinguish between monomeric, oligomeric, and higher-order assemblies across diverse biological systems, particularly where large aggregates or complexes represent the most functionally relevant species. Consistent with this broader concept, a previously reported digital seed amplification assay detected low-concentration α-synuclein aggregates and used brightness and eccentricity measurements to extract aggregate growth and morphology information,^21^ supporting the potential of digital compartmentalized assays in aggregate characterization beyond binary counting.

## Conclusion

In conclusion, brightness-based Simoa profiling provides a simple and accessible means of extracting additional morphological information from an established ultrasensitive detection platform without requiring modification of assay chemistry or instrumentation. Given the widespread use of Simoa in biomarker development and protein quantification, this approach may enable interrogation of aggregate size distributions across a broad range of existing assays and disease contexts. Future optimization of assay design, calibration strategies, and analytical pipelines may further improve the sensitivity and resolution of brightness-based measurements, expanding their utility for studying aggregate heterogeneity and disease-associated changes in protein aggregation. More broadly, this work highlights the potential of digital single-molecule assays to provide morphological as well as quantitative information, extending their utility beyond conventional analyte detection.

## Supplementary Material



## Data Availability

All original brightness analysis code has been deposited at Zenodo at DOI: 10.5281/zenodo.20140389 and is publicly available as of the date of publication. All data are available in the manuscript or the Supporting Information. Raw data available on written request to D.K.

## Notes

Additional experimental details and methods, including detailed patient information and supporting experimental data (PDF)
