# Supplementary material for: Profiling Protein Aggregate Size Using Single-Molecule Array Technology
Source: Anal Chem. 2026 Jun 25;98(26):19882–93. doi: 10.1021/acs.analchem.6c03315 (PMC13347706; doi:10.1021/acs.analchem.6c03315)
Supplement: Supplementary file 1 [file ac6c03315_si_001.pdf]

SUPPLEMENTARY INFORMATION

Profiling protein aggregate size using single-molecule array technology

Dorothea Böken[1,2,#], Yunzhao Wu[1,2,#], Jianli Zhang[3], Zengjie Xia[4,5,6], Paula Beltran-Lobo[7], Cara L. Croft[8], Savinu Weerasekera[1], Amanda Heslegrave[4,5], Henrik Zetterberg[4,5,6,9,10,11,12], Ashvini Keshavan[5,13], Jonathan M. Schott[5,13], Maria Jimenez-Sanchez[7], David C. Duffy[3], David Klenerman[1,2*]

[1] Yusuf Hamied Department of Chemistry, University of Cambridge, Cambridge, CB2 1EW, United Kingdom

[2] UK Dementia Research Institute, University of Cambridge, Cambridge, CB2 0AH, United Kingdom

[3] Quanterix Corporation, 900 Middlesex Turnpike, Billerica, Massachusetts, 01821, United States of America

[4] UCL Queen Square Institute of Neurology, London, WC1N 3BG, United Kingdom

[5] UK Dementia Research Institute at University College London, London, WC1N 3BG, United Kingdom

[6] Hong Kong Center for Neurodegenerative Diseases, Hong Kong, 999077, China

[7] Department of Basic and Clinical Neuroscience, Maurice Wohl Clinical Neuroscience Institute, King's College London, London, SE5 9RX UK

[8] Centre for Neuroscience, Surgery and Trauma, The Blizard Institute, Queen Mary University of London, London, E1 2AT UK

[9] Department of Psychiatry and Neurochemistry, Institute of Neuroscience and Physiology, The Sahlgrenska Academy at the University of Gothenburg, Mölndal, 43180, Sweden

[10] Clinical Neurochemistry Laboratory, Sahlgrenska University Hospital, Mölndal, 43180, Sweden

[11] Wisconsin Alzheimer's Disease Research Center, University of Wisconsin School of Medicine and Public Health, University of Wisconsin–Madison, Madison, Wisconsin, 53792, United States

[12] Department of Pathology and Laboratory Medicine, University of Wisconsin School of Medicine and Public Health, Madison, Wisconsin, 53792, United States

[13] Dementia Reseach Centre, Department of Neurodegenerative Disease, UCL Queen Square Institute of Neurology, London, WC1N 3BG, United Kingdom

# These authors contributed equally to this work.

* Corresponding author: dk10010@cam.ac.uk

Table of Contents

Supplementary Table S1	S3
Supplementary Table S2	S4
Supplementary Table S3	S5
Supplementary Figure S1	S6
Supplementary Figure S2	S7
Supplementary Figure S3	S8

Supplementary Table S1. Sequence and lower limits of detection (LLOD) of the peptides used in this study.

Peptide	Sequence	LLOD [pM]
'Monomer'	GAAPPGQKGQ(PEG)$_4$GKPQPAGAQG	-
'Dimer'	GAAPPGQKGQ(PEG)$_4$GAAPPGQKGQ	478.90
'Trimer'	GAAPPGQKGQ(PEG)$_6$GAAPPGQKGQ(PEG)$_6$GAAPPGQKGQ	67,10
'Tetramer'	GAAPPGQKGQ(PEG)$_6$GAAPPGQKGQ(PEG)$_6$GAAPPGQKGQ (PEG)$_6$GAAPPGQKGQ	16,27
'Pentamer'	GAAPPGQKGQ(PEG)$_6$GAAPPGQKGQ(PEG)$_6$GAAPPGQKGQ (PEG)$_6$GAAPPGQKGQ(PEG)$_6$GAAPPGQKGQ	1,66
SiNaP 15nm	KK(PEG)$_8$GAAPPGQKGQ	1,12
SiNaP 30nm	KK(PEG)$_8$GAAPPGQKGQ	0,13

Supplementary Table S2. Patient information brain homogenate.

Patient	Sex	Age [years]	Braak Stage	Region	BBN
**AD1**	Female	72	VI	BA6/8	BBN001.36924
**AD2**	Male	75	VI	BA6/8	BBN001.37400
**AD3**	Female	85	VI	BA6/8	BBN_25739
**AD4**	Male	75	VI	BA6/8	BBN001.36839
**AD5**	Male	75	VI	BA6/8	BBN001.36689
**HC1**	Female	71	0	BA6/8	BBN001.29882
**HC2**	Male	72	0	BA6/8	BBN001.30178
**HC3**	Female	73	0	BA6/8	BBN001.35138
**HC4**	Male	71	0	BA6/8	BBN001.30916
**HC5**	Male	82	0	BA6/8	BBN001.35549

Supplementary Table S3. Patient information Wolfson CSF study plasma samples.

Diagnosis	Sex	Age [years]	Number of patients
AD	Female	63.9 ± 6.2	66
	Male	63.8 ± 6.7	63
Non-AD	Female	60.7 ± 7.7	38
	Male	64.0 ± 7.7	86

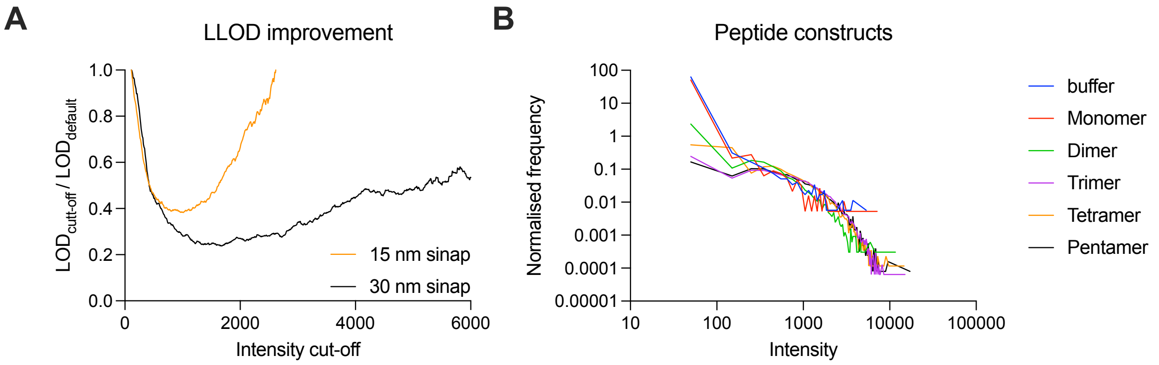

Supplementary Figure S1. Effect of brightness thresholding and construct-dependent intensity profiles. (A) Effect of increasing brightness thresholds on detection sensitivity for silica nanoparticle-based constructs. Applying higher intensity cut-offs (i.e. restricting analysis to higher-intensity microwells. **(B)** Brightness distributions for HT7 epitope peptide constructs containing one to five epitopes.

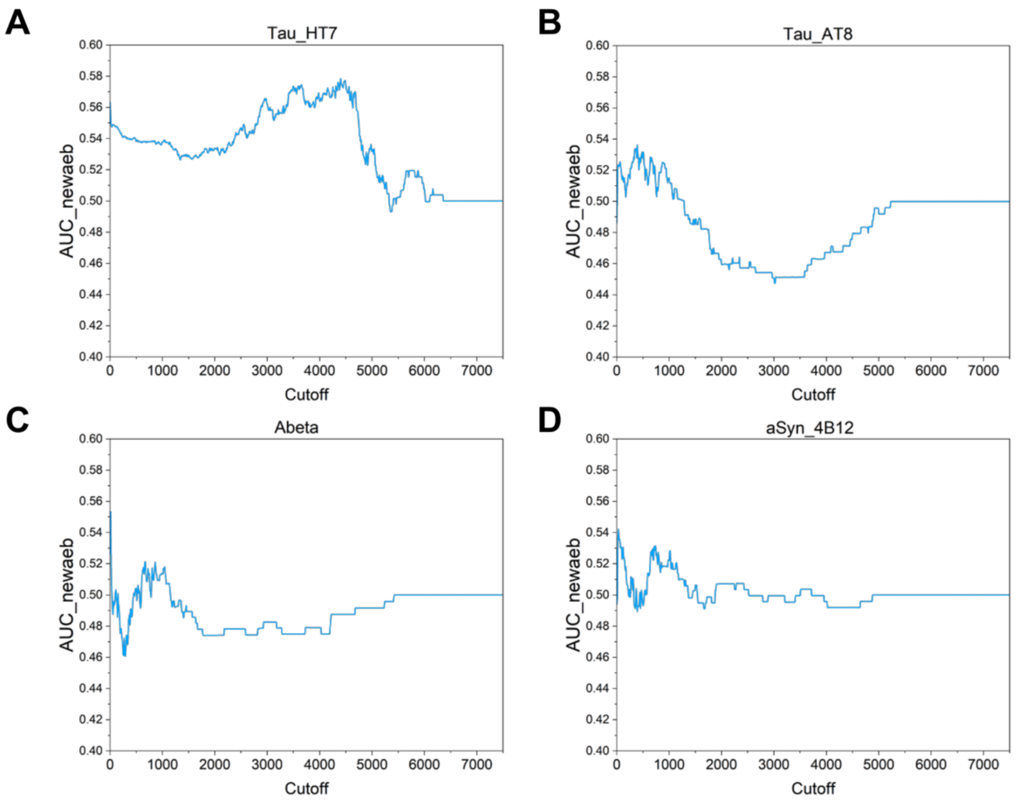

Supplementary Figure S2. Diagnostic performance across brightness thresholds for aggregate detection in blood. Area under the ROC curve (AUC) as a function of increasing brightness cut-off for Simoa-based aggregate detection in blood samples, shown for assays targeting **(A)** total tau (HT7), **(B)** phosphorylated tau (AT8), **(C)** amyloid-β (Aβ), and **(D)** α-synuclein (αSyn) aggregates.

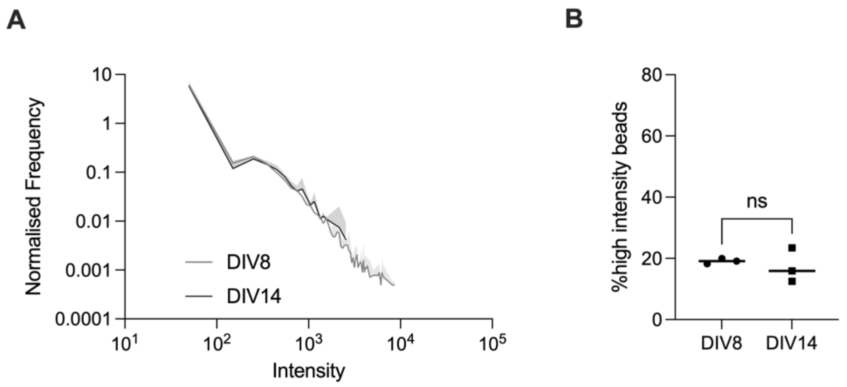

Supplementary Figure S3. Brightness analysis of tau aggregates in media from untransduced control neurons. (A) Brightness distributions of tau aggregates in conditioned media from untransduced primary mouse neurons at different timepoints (DIV8 and DIV14). Data represent mean ± SD from *n* = 3 biological replicates. **(B)** Percentage of bright aggregates (well intensity >1000). Welch's t-test, ns: $p > 0.05$.
